# Uptake and barriers to cervical cancer screening among human immunodeficiency virus-positive women in Sub Saharan Africa: a systematic review and meta-analysis

**DOI:** 10.1186/s12905-023-02479-w

**Published:** 2023-06-27

**Authors:** Meresa Berwo Mengesha, Tesfaye Temesgen Chekole, Hagos Degefa Hidru

**Affiliations:** 1 Department of Midwifery, College of Medicine and Health Science, Adigrat University, Adigrat, Tigray Ethiopia; 2 Department of Public Health, PhD Fellow at Dilla University, Dilla, Ethiopia; 3 Department of Public Health, College of Medicine and Health Science, Adigrat University, Adigrat, Tigray Ethiopia

**Keywords:** Cervical cancer, Screening, Uptake, Barriers, Sub-Saharan, Africa

## Abstract

**Background:**

Cervical cancer is the leading cause of disability and mortality among women in Africa. Despite a significant correlation between HIV/AIDS and cervical cancer, there is unacceptably low coverage of the uptake of cervical cancer screening among human immunodeficiency virus-positive women in Sub-Saharan Africa. Individual primary studies are limited in explaining the patterns of uptake of cervical cancer screening. This review therefore considers the uptake of cervical cancer screening and its barriers among human immunodeficiency virus-positive women in Sub-Saharan Africa.

**Methods:**

We systematically searched articles published until December ^31^, 2019, from the PubMed, Cochrane Library, POP LINE, Google Scholar, African Journals Online and JURN databases. The quality of the included articles was assessed by using the Newcastle‒Ottawa Scale, and the coverage of uptake of cervical cancer screening was pooled after checking for heterogeneity and publication bias. The random effect model was used, and subgroup analysis estimates were performed by country.

**Results:**

Twenty-one studies comprising 20,672 human immunodeficiency virus-positive women were included. Applying a random effect model, the overall cervical cancer screening uptake among this group of women in Sub-Saharan Africa was estimated to be 30% (95% CI: 19, 41, I^2^ = 100%). The main barriers to uptake of cervical screening include poor knowledge about cervical cancer and screening, low risk perception of cervical cancer, fear of test result and fear of screening as painful, lack of access to screening services, high cost of screening service, and poor partner attitude and acceptance of the service. The perception of an additional burden of having a cervical cancer diagnosis was found to be a unique barrier among this population of women.

**Conclusion:**

The unacceptably low coverage of uptake of cervical cancer screening would indicate that the need to scale up the opportunities to these groups of women as well. This review revealed that in addition to structural and health care system barriers, sociocultural and personal barriers are powerful barriers in HIV-positive women. For these cohorts of population, a particular obstacle was discovered to be perception of an additional burden of having cervical cancer.

**Supplementary Information:**

The online version contains supplementary material available at 10.1186/s12905-023-02479-w.

## Introduction

Cervical cancer was the fourth most common cancer in women, ranking after breast cancer, colorectal Cancer and lung cancer and continues to become a major cause of death in resource limited countries such as Sub Saharan Africa [1, 2]. The incidence of cervical cancer in sub-Saharan Africa (SSA) ranges from 43.3/100,000 to 69.8/100,000 women and is 15 times higher than that in developed Nations [1–3]. Projections suggest that around 1.27 million people will be diagnosed with cancer each year in Africa annually by 2030.From these, cervical cancer accounts 80,400 cases [4].

As the US Centers for Disease Control and Prevention (CDC) categorized cervical cancer as an acquired immunodeficiency syndrome (AIDS) defining cancer in 1970, the coinfection of the two diseases represents substantial public health impacts in SSA [5–7]. Infection with HIV exponentially increases women’s risk of cervical intraepithelial lesions and cervical cancer [8–11]. Although the association is obscure, it is believed to be due to high viral load and to challenges in adherence of anti-retroviral drugs in the region, which may impair the cell-mediated immune response [12–14]. Due to the HIV epidemic in SSA, the relationship between HIV/AIDS and cervical cancer can result in significant individual and health system burdens [7].

According to the recommendation of the CDC, National Institute of Health (NIH), and HIV Medicine Association (HIVMA), HIV-infected women should undergo two Pap tests within the first year after the diagnosis of HIV infection, followed by annual Pap testing irrespective of their age [11, 15]. The World Health Organization (WHO) has established guidelines on cervical cancer screening and follow-up for HIV-positive women living in developing countries [16].

WHO recommends that women living in HIV high prevalence areas and be sexually active; as soon as possible to initiate screening for cervical cancer [17]. Although these recommendations are deemed in place, cervical cancer screening in developing countries is lower, and in sub-Saharan Africa, the screening coverage ranges from 0.4% to 20.2%, and the screening coverage is greater than 60% in developed countries [18, 19].

The incidence of co-infection HIV/AIDS with cervical cancer is high globally and in SSA [20–24].

Although the reasons for not screening for cervical cancer vary from region to region, HIV-positive women have unique challenges that hinder them from utilizing the service. However, in most cases, they share the same barriers with HIV-negative women [25, 26]. This review would summarize the barriers/unique to the low coverage of cervical cancer screening among this high group of women in SSA countries.

Although there have been a few systematic review studies addressing the barriers to cervical cancer screening in the whole population in Sub-Saharan Africa (SSA), there is a lack of evidence on the pooled effect (meta-analysis) of uptake and barriers to CC (Cervical Cancer) screening among this group of women (HIV-positive women) in SSA. Therefore, this review aimed to describe the ranges of and barriers to cervical cancer screening among HIV-positive women (unique barriers) in SSA. Uptake of cervical cancer screening for this review is defined as those respondents who have been screened for cervical cancer once in their lifetime [19, 27, 28]. This review summarizes studies on the uptake of cervical cancer screening among HIV-positive women in SSA to generate comprehensive data for health professionals and policy makers in that area to support secondary prevention services for cervical cancer.

## Methods

### Data sources and search strategies

This review was written in accordance with the recommendation of the Preferred Reporting Items for Systematic Review and Meta-Analysis (PRISMA-P) 2015 statement [29]. Observational studies published in SSA countries and written in English language and all published articles up to December 31^st^, 2019 were searched systematically and thoroughly in the following databases and search engines: PubMed, Cochrane Library, POPLINE, Google Scholar, African Journals Online (AJOL) and JURN. In addition, a back and forth review of references from eligible included studies was undertaken.

#### Key terms/phrases employed search strategy in PubMed

“(((((uptake) OR (("Facilities and Services Utilization"[Mesh])))))) AND "Uterine Cervical Neoplasms"[Mesh]) AND ((((("Mass Screening"[Mesh])) AND ("Early Detection of Cancer"[Mesh]) AND "Diagnosis"[Mesh]) AND "diagnosis" [Subheading] ))) AND (("Africa"[Mesh]) OR "Africa South of the Sahara"[Mesh])”.

#### Inclusion criteria

Observational studies that reported uptake of CC screening among HIV-positive women, measured by those respondents who screened for CC at least once in their lifetime, studies conducted in SSA, all published articles in English language, and no restriction in the publication year were included.

#### Exclusion criteria

Excluded studies report populations other than HIV-positive women, unable to access full text, studies did not address the barriers to cervical cancer screening services, and studies published in languages other than English.

The primary outcome of this review was to determine a pooled single estimate of the uptake of cervical cancer screening among HIV-positive women in SSA, and the secondary aim was to identify the barriers to CC screening services among HIV-positive women in SSA. For this review, any documented evidence of having screened and become involved in mass/campaign screenings, clinic/facility-based or programmatic screenings at least once in life is considered uptake of CC screening and is similar to the barriers to screening.

### Data extraction

Initially, an advanced and systematic search was made via the identified databases. In addition, a manual review of references of included studies was conducted to access additional relevant articles. Next, studies published other than in English language, conducted out of SSA; unrelated and irrelevant articles based on their title and abstract were excluded. Then, the remaining articles were imported into Endnote version 7, and duplicate articles were removed. After exact duplicates were removed, two reviewers (MBM and HDH) independently conducted abstract and full text reviews to determine the articles to be included and excluded. In the case of disagreement, divergent views were resolved by consulting a third reviewer TTC. The final articles selected were then reviewed by the two reviewers for data abstraction. Finally, data were extracted from the included studies through a structured data extraction form and presented using tables: name of authors, study period, year of publication, study design, country of the study, residence, type of screening for cervical cancer, total sample size, uptake of cervical cancer screening (proportion), reasons for not being screened, and frequency/number of identified barriers [frequency/number of women who reported barriers among those who did not undergo cervical cancer screening in their lifetime].

### Risk of bias assessment

The quality assessment of cohort and cross-sectional studies was assessed using the Newcastle‒Ottawa Scale (NOS) [30, 31]. The NOS included 3 categorical criteria comprising a maximum score of 9 points. The quality of the included studies was rated according to the following scale: ≥ 7 was considered good, 3 to 6 fair and < 3 poor. For the purpose of this study, we included studies with fair to good quality.

### Strategy for data synthesis

Coverage (proportion) of uptake of cervical cancer screening and estimates of barriers gained from each study were pooled and estimated as a single estimate. The heterogeneity level across the studies was tested using the Higgins test, where the I^2^ statistics were determined and reported. Publication bias was assessed using a visual inspection of funnel plots and Egger’s regression test. During meta-analysis, in the case of a high level of heterogeneity, a random effect model was used, and a subgroup analysis estimate was performed by the countries in which the study was performed.

Sensitivity analysis was performed for studies included in the review. The meta-analysis, subgroup analysis, sensitivity analysis and publication biases were carried out with statistical R-software version 3.6.1 and Stata Version 14 for sensitivity analysis. Sixteen variables, name of authors, study period, year of publication, study design, country of the study, residence, type of screening for cervical cancer, total sample size, uptake of cervical cancer screening (proportion), reasons for not being screened, and frequency of identified barriers, were entered into statistical R-software version 3.6.1. to run the meta-analysis. In addition to the use of a random effect model, sensitivity analysis was performed in Stata 14 ‘influence-analysis (metaninf) tests, which indicated that the estimated values of all the included studies were within the CI. Accordingly, we declared that two studies affected the results of the other studies. Nevertheless, they were not as influential and did not significantly affect the result. The protocol of this systematic review is under registration process with the prospective registration number of 167,569 for systematic reviews (PROSPERO acknowledgement of receipt [167569]).

## Results

### Study selection

**Fig. 1:**
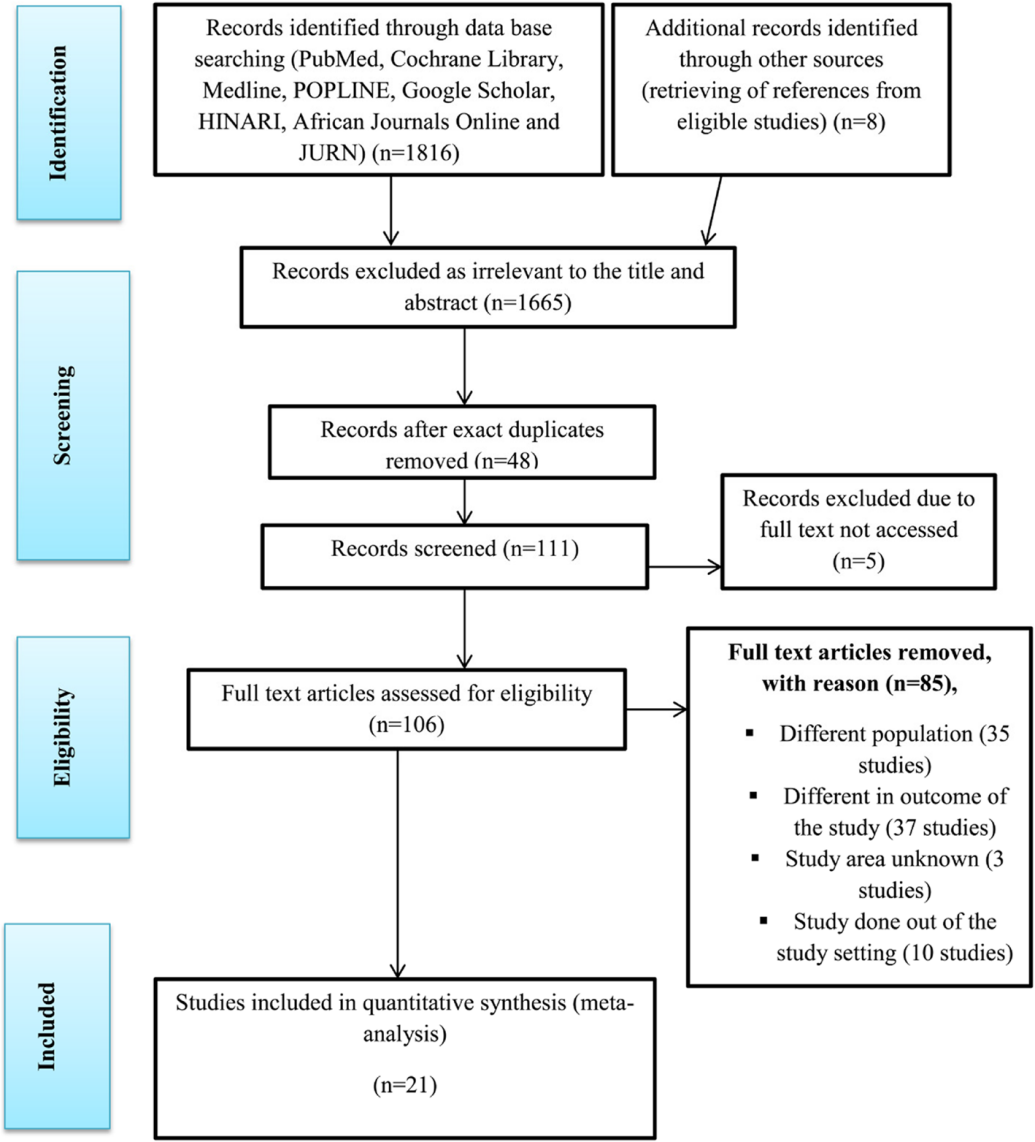
PRISMA statement presentation for systematic review and meta-analysis of the uptake of cervical cancer screening among HIV-positive women in Sub-Saharan African countries

The online search yielded 1824 records through database searching. From this search, 1803 retrieved studies were removed through a step-by-step process for the following reasons: 1665 were excluded as irrelevant by title and abstract, 48 were removed as duplicates, 5 full texts were not accessible, 35 reflected different study populations, 37 reflected different outcomes, 3 had unknown study areas, and 10 were out of the study setting. One hundred and six full text articles were assessed for eligibility, and 21 articles were included in the quantitative synthesis (meta-analysis) (Fig. 1). Finally, the quality of the included articles was assessed by using NOS criteria (Table 1) and discussed in detail in the Annex (Tables S1 and S2).

**Table 1:** Characteristics of included studies in the systematic review and meta-analysis performed on the uptake of cervical cancer screening among HIV-positive women in Sub-Saharan Africa. (*N* = 21)

Author, year, Ref	Country	Residence	Study design/setting	Nature of study and screening	Types of screening	Sample size	Uptake of CC screening	Quality Score (NOS)
Belete N. et al.2015 [32]	Ethiopia	Urban	C/S QN-QL	Population based, clinic based screening	pap smear	322	11.5	8
Ashagrie A. et al.2017 [33]	Ethiopia	rural‒urban	C/S QN	Population based, clinic based	VIA/VILI	311	16.1	8
Solomon K. et al.2019 [34]	Ethiopia	rural‒urban	C/S QN-QL	Population based, clinic based	VIA/VILI	475	25	8
Cholli P. et al.2017 [35]	Cameron	rural‒urban	Cohort QN	Programmatic, mass screening	VIA/VILI	1170	79.7	9
Pediatric A. et al.2019 [36]	Côte d’Ivoire	Urban	cohort QN	Population based, clinic based	VIA/VILI	1991	40.3	9
Horo A. et al.2012 [37]	Côte d’Ivoire	rural‒urban	C/S QN	Population based, clinic based	VIA/VILI	1913	59.7	7
Nega A. et al.2018 [38]	Ethiopia	Rural	C/S QN	Population based, clinic based	VIA/VILI	496	10	8
Assefa A. et al.2019 [39]	Ethiopia	Urban	C/S QN	Population based, clinic based	VIA/VILI	342	40.1	8
Wanyenze RK. et al. 2011 [40]	Uganda	Urban	C/S QN	Population based, clinic based	VIA/VILI	520	56	7
Mtengezo J.2019 [41]	Malawi	rural‒urban	C/S QN-QL	Population based, clinic based	VIA/VILI	291	27.8	8
Ezechi OC.et al.2013 [42]	Nigeria	Urban	C/S QN	Population based, clinic based	VIA/VILI	1517	9.4	9
Mokhele I. et al.2016 [43]	South Africa	Urban	C/S QN	Population based, clinic based	pap smear	1202	57.2	8
Wanyenze R.et al.2017 [44]	Uganda	rural‒urban	C/S QN	Population based, mass screening	VIA/VILI	5198	30.3	9
Lukorito J.et al.2017 [45]	Kenya	rural‒urban	C/S QN	Population based, clinic based	VIA/VILI	306	19	7
Gundani HV.et al.2013 [46]	Zimbabwe	Urban	C/S QN	Simple survey, clinic based	pap smear	70	11.4	3
Matangaidze O.2015 [47]	Zimbabwe	Urban	C/S QN-QL	Population based, clinic based	VIA/VILI	208	8.7	6
Shiferaw S. et al.2016 [48]	Ethiopia	Urban	C/S QN-QL	Population based, clinic based	VIA/VILI	594	10.8	8
Deribe L.2018 [49]	Ethiopia	Rural‒urban	C/S QN	Population based, clinic based	Pap smear	357	7.8	8
Erku DA. et al. 2017 [50]	Ethiopia	Rural‒urban	C/S QN	Population based, clinic based	Pap smear	302	23.5	7
Kemper KE.2019 [51]	Kenya	Rural‒urban	C/S QN	Population based, mass screening	VIA/VILI	3007	56	9
Bulto G. e tal.2019 [52]	Ethiopia	Rural‒urban	C/S QN	Population based, clinic based	VIA	423	2.1	8

VIA visual inspection with acetic acid, *VILI* visual inspection with Lugol's iodine, *NOS* Newcastle Ottawa Scale, *C/S* cross-sectional, *QN* quantitative, *QL* qualitative, *QN-QL* quantitative‒qualitative

### Study characteristics

In the review, there were nineteen [19] cross-sectional studies and two (2) cohort studies, of which 21 had fair to good quality, containing (*n* = 21,015) HIV-positive women. The smallest sample size was a study performed in Zimbabwe with 70 participants, [46] while the largest study was performed in Uganda [44] and included 5198 individuals. The mean sample size for the included studies was 1001. The lowest coverage of cervical cancer screening uptake was estimated by Bulto G. et al. [52] at 2.1%, while the highest coverage was estimated by Cholli P. et al. [35] at 79.7%. There were 9 studies from Ethiopia [52], and the remainder were from Kenya [45, 51], Zimbabwe [46, 47], Côte d’Ivoire [36, 37], Uganda [40, 44], Cameron [35], Nigeria [42], Malawi [41] and the Republic of South Africa [43]. Twelve of the included studies were conducted in both rural‒urban settings simultaneously. Related to the nature and type of screening, 1 programmatic study, 19 population-based studies and 1 none of them and 3 of the study mass screenings were conducted, and clinic-based screening was performed in 18 of the studies. With respect to the type of screening, 16 of the studies used visual inspection with acetic acid (VIA) and visual inspection with Lugol's iodine (VILI), and 5 of the studies used Pap smears (Table 1). Regarding the barriers to CC screening, 15 of the studies were identified by quantitative methods, 5 included both quantitative and qualitative methods, and one study was identified by a qualitative approach only (Table 2).

**Table 2:** Barriers to cervical cancer screening uptake among HIV-positive women in Sub-Saharan Africa by article type and frequency

Barriers to Cervical Cancer Screening	Frequency of identified barrier for uptake of CC	Frequency of women not screened for cervical cancer	Both qualitative and quantitative study	Quantitative study	Qualitative study	Number of studies identified
Lack of financial/high cost of the test	36	285	Belete N. et al. 2015 [32]	-		9
	1	261	-	Ashagrie A.et al. 2017 [33]		
	80	803	-	Pediatric A. et al.2019 [36]		
	2	205	-	Assefa A. et al.2019 [39]		
	99	291	Mtengezo J.2019 [41]	-		
	108	1374	-	Ezechi OC.etal.2013 [42]		
	15	248	-	Lukorito J.etal.2017 [45]		
	33	531	Shiferaw S.2016 [48]	-		
	64	231	-	Erku DA. et al. 2017 [50]		
No good attitude and acceptance of partner	12	285	Belete N. et al. 2015 [32]	-		6
	1	261	-	Ashagrie A.etal.2017 [33]		
	38	1374	-	Ezechi OC.etal.2013 [42]		
	56	375	Solomon K. et al.2019 [34]	-		
	19	531	Shiferaw S. etal. 2018 [54]	-		
	8	231	-	Erku DA. et al. 2017 [50]		
Fear of test result as positive	37	285	Belete N. et al. 2015 [32]	-	Bukriwa A. etal.2015[53]	8
	2	261	-	Ashagrie A. etal.2017 [33]		
	177	803		Pediatric A. et al.2019 [36]		
	105	375	Solomon K. et al.2019 [34]	-		
	33	205	-	Assefa A. et al.2019 [39]		
	31	531	Shiferaw S. et al.2016 [48]	-		
	164	231	-	Erku DA. et al. 2017 [50]		
Do not know where the place for screening is performed	4	261	-	Ashagrie A. etal.2017 [33]		4
	13	205	-	Assefa A. et al. 2019 [39]		
	50	291	Mtengezo J..2019 [41]	-		
	117	531	Shiferaw S. et al.2016 [48]	-		
Fear of screening and procedure is painful	5	261	-	Ashagrie A. etal.2017 [33]	Bukriwa A. etal.2015 [53]	8
	13	1374	-	Ezechi OC. etal.2013 [42]		
	37	375	Solomon K. et al.2019 [34]	-		
	376	3637	-	Wanyenze RK. et al. 2011 [40]		
	8	190	Matangaidze O.2015 [47]	-		
	32	531	Shiferaw S. et al.2016 [48]	-		
	159	231	-	Erku DA. et al. 2017 [50]		
Perception of I am not sick	69	261	-	Ashagrie A. etal.2017 [33]	Bukriwa A. etal.2015 [53]	6
	75	375	Solomon K. et al.2019 [34]	-		
	66	291	Mtengezo J.2019 [41]	-		
	212	531	Shiferaw S. et al.2016 [48]	-		
	205	231	-	Erku DA. et al. 2017 [50]		
Long waiting time of the procedure	1	261	-	Ashagrie A. etal.2017 [33]	Bukriwa A. etal.2015 [53]	8
	39	1374	-	Ezechi OC. etal.2013 [42]		
	913	3637	-	Wanyenze RK. et al. 2011 [40]		
	8	190	Matangaidze O.2015 [47]	-		
	49	291	Mtengezo J.2019 [41]	-		
	43	285	Belete N. et al. 2015 [32]	-		
	45	531	Shiferaw S. et al.2016 [48]			
	44	231		Erku DA. et al. 2017 [50]		
Lack of screening facility and nearest facility	3	261	-	-Ashagrie A. etal.2017 [33]		Six studies (6)
	501	3637	-	Wanyenze RK. et al. 2011 [40]		
	8	190	- Matangaidze O.2015 [47]	-		
	50	291	Mtengezo J.2019 [41]	-		
	25	531	Shiferaw S. et al.2016 [48]			
	87	231		Erku DA. et al. 2017 [50]		
Lack of knowledge about cervical cancer and screening	1059	3637	-	Wanyenze RK. et al. 2011 [40]	Bukriwa A. etal.2015 [53]	8
	154	190	Matangaidze O.2015 [47]	-		
	434	803	-	Pediatric A. et al.2019 [36]		
	137	205	-	Assefa A. et al.2019 [39]		
	26	375	Solomon K. et al.2019 [34]	-		
	34	62	-	Gundani HV. et al.2013 [46]		
	6	531	Shiferaw S. et al.2016 [48]	-		
Incompetent and inadequate knowledge of health care provider	1	261	-	Ashagrie A. etal.2017 [33]		4
	120	803	-	Pediatric A. et al.2019 [36]		
	16	291	Mtengezo J.2019 [41]	-		
	8	231	-	Erku DA. et al. 2017 [50]		

*QL* Qualitative, *QN* Quantitative, *CC* Cervical Cancer

### Synthesized outcomes

As shown by the I^2^ statistics, evidence of high heterogeneity was observed between the studies. Evidence of publication bias was also observed in this systematic review and meta-analysis through visual inspection of an asymmetrical funnel plot; however, the quantitative Egger’s linear regression test *P* value (0.69) revealed insignificant publication bias.

**Fig. 2:**
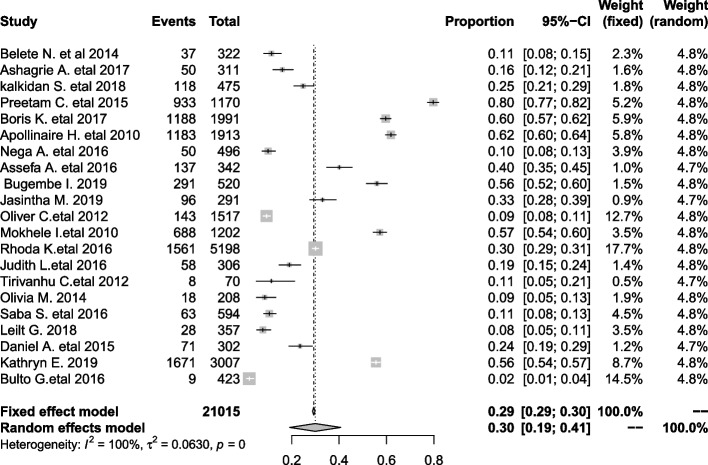
Pooled proportion of uptake of CC screening among HIV-positive women in SSA

**Fig. 3:**
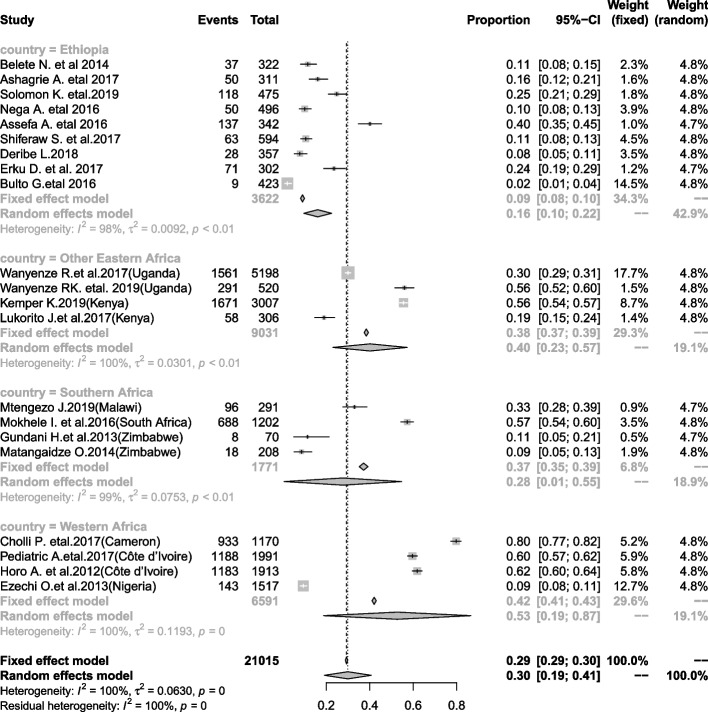
Forest plot presenting subgroup analysis of uptake of CC screening by country

**Fig. 4:**
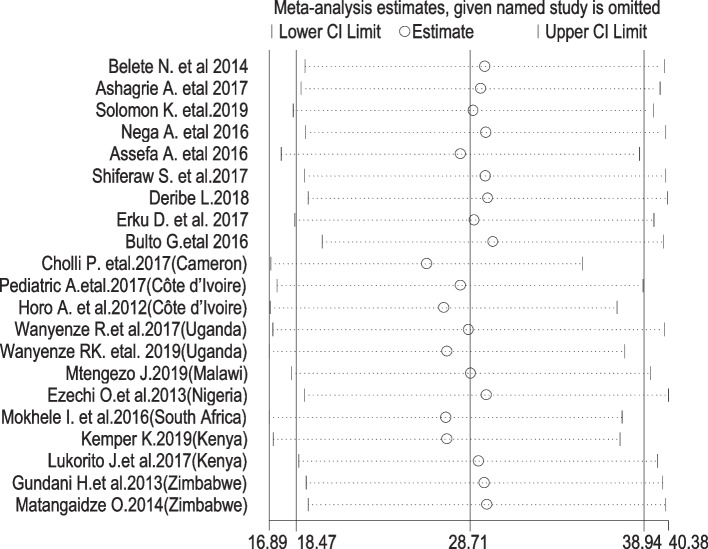
Sensitivity analysis for studies included in a systematic review and meta-analysis of the uptake of cervical cancer screening among HIV-positive women in Sub-Saharan Africa

In SSA, CC screening uptake was estimated to be 30% (95% CI: 19, 49, I^2^ = 100%) according to pooling estimates of included primary studies conducted in different parts of SSA (Fig. 2). Due to significant heterogeneity between the included studies, we conducted meta-regression to determine the possible source of heterogeneity. Accordingly, the country where the study occurred was found as a possible source and explained 21.16% of the observed heterogeneity. We did and reported pooled estimates of uptake of CC screening through subgroup analysis of countries (i.e., western Africa, southern Africa, other eastern Africa and Ethiopia). In the subanalysis, the highest coverage of estimated pooled uptake of CC screening was 53% (95% CI: 19, 87) in western Africa, 40% (95% CI: 23, 57) in other eastern African countries other than Ethiopia compared to pooled data of sub-Saharan Africa (this review), 30% (95% CI: 19, 49, I^2^ = 100%), and southern Africa, 28% (95% CI: 10, 55). The lowest coverage of uptake of CC screening among HIV-positive women was in Ethiopia at 16% (95% CI: 10, 22) (Fig. 3). We performed a sensitivity test and found that two studies [41, 44] affected the results of the other studies. Nevertheless, they were not as influential and did not significantly affect the result (Fig. 4).

### Barriers to uptake of cervical cancer screening (quantitative synthesis)

The most common reasons for not participating in CC screening among HIV-positive women were lack of knowledge about cervical cancer and screening 49% [95% CI: 29, 68], perceived risk of cervical cancer (perception of I am healthy) 23% [95% CI: 19, 26], fear of test result as positive 18% [95% CI: 7, 30], and fear of screening and procedure is painful 11% [95% CI: 5, 17] (Table 3).

**Table 3:** Barriers to the uptake of cervical cancer screening among HIV-positive women in SSA, meta-analysis (random effect model)

Barriers	Number of Included Studies	Pooled estimate %, (95% CI)	*P* value	*I*^2^
Lack of finances/high cost of testing	9	10.00 [5.00, 14]	< 0.01	98
Negative attitude and nonacceptance of partner	6	5 [2,5]	< 0.01	96
Fear positive test result	8	18 [7,30]	< 0.01	99
Do not know where screening is performed	4	12 [6, 17]	< 0.01	88
Fear of screening and procedure as painful	8	11 [5, 17]	< 0.01	99
Perception of “I am healthy”	6	23 [19, 26]	< 0.17	44
Long waiting time for the procedure	8	11 [2, 19]	< 0.01	100
Lack of screening facility and nearby facility	6	9 [2, 15]	< 0.01	98
Lack of knowledge about CC and screening	8	49 [29, 68]	< 0.01	99
Incompetent and inadequate knowledge of health care providers	4	7 [0.00, 16]	< 0.01	98

### Barriers to uptake of cervical cancer screening (qualitative)

#### Knowledge about cervical cancer and screening

Poor knowledge of CC and poor awareness of CC screening were identified as the main reasons for not undertaking screening (barrier) in three studies contributing qualitative results (Table 2). In one study, a participant reported “Handizivi (literally meaning I don’t know)” [47]. Most HIV-positive respondents recognized that cervical cancer can be prevented, but all of them did not believe the disease can be effectively treated once diagnosed [28, 54].

### Risk perception of cervical cancer

Although most HIV-positive women perceived a high risk of getting the disease, some of them would not participate in CC screening because of the perception of “I am not sick or I do not have signs and symptoms of the disease” [28, 47, 48, 53]. The statement by on HIV-positive women summarized the potential lack of insight into risk with her comment that “I have not truly had signs to show me that I might have it” [53].

### Fear of test results and fear of screening procedures

Four studies revealed that fear of a positive test result was a potential hindrance for not using the service. Belete N. et al. 2015 [32] reported that one participant stated, “the word you have a cancer diagnosis is truly irritating beside my HIV, I think I will get hopeless, if I am diagnosed as having cervical cancer.” HIV-positive women were scared to screen for any disease including cervical cancer because they were frightened to add another stressful issue that would scare them, and this disease comes with misconceptions of the need to remove organs (ovary, womb) [34, 53, 54]. Fear of pain related to screening was also identified as a barrier for utilization of the service in four studies [34, 47, 48, 53].

### Partner attitude and acceptance of the service

In most SSA countries, there is a male-dominated family model, with the husband deciding every issue of the family as the primary income earner. Hence, the male partner’s reluctance to have their wife undergo CC screening is often related to the belief that this procedure violates the pride and privacy of their partner [32, 34, 48].

### Access to screening services

Lack of well-equipped health facilities and qualified personnel to carry out the procedure were identified as barriers for accessibility and uptake of CC screening. Trouble in navigating health care facilities and lack of awareness about the location of screening facilities were also reported as barriers [41, 47, 54]. Waiting times for screening and length of the procedure were also given as potential reasons for nonuptake of the service [34, 47, 53, 54].

### Cost of screening and finance

Four studies reported that a lack of financial support and associated costs for screening were barriers to the uptake of CC screening. In areas where poverty is prevalent, payment for nonemergency health services such as CC screening is a challenge. In addition, the cost of transportation contributes to the nonaccessibility and nonuse of the service [32, 41, 54].

## Discussion

Cervical cancer is a major cause of disability and mortality and has a substantial health impact in SSA. The scale up of cervical cancer screening coupled with HPV vaccination can reduces cervical cancer disease and has a potential to make cervical cancer disease no more threat to the coming generation [16]. The coverage of CC through screening in developing countries compared to developed countries is very low, covering less than 20% [18]..

This systematic review and meta-analysis found that the pooled coverage of uptake of CC screening among HIV-positive women in SSA was 30% [95% CI: 19, 41]. This finding was comparable with findings reported from Tigray, Ethiopia, 19.8% [55], but lower than findings estimated from within United States urban HIV infected women at 44% (US), Netherlands, 69.54% [56], Qatar, 40% [57], and Republic of South Africa, 52% [58]. With the exception of the United States study, the observed discrepancy might be due to research on the whole population, thus not necessarily recognizing that HIV-infected women may have unique challenges. Variations have been shown to be due to differences in health care systems, access to reproductive health services, implementation of CC screening programs among HIV positive groups, knowledge of CC and screening, community mobilization as well as awareness of the association of the two diseases. This implies that monitoring and evaluation of cervical screening uptake, scaling up of the service, widespread coverage of HPV vaccination and sustainability of a cervical cancer prevention programs has a paramount importance in reducing cervical cancer disease in resource limiting areas such as Sub Saharan Africa. Moreover, screening service should be incorporated to the routine care and treatment services..

The findings from the subanalysis showed that the pooled coverage of uptake of CC screening among HIV-positive women was higher in western Africa and other Eastern parts of African countries other than Ethiopia. The observed differences might be due to the implementation of CC screening programs among HIV-positive women in these regions, sociodemographic differences, and the health care system.

Considering this low coverage of cervical cancer screening uptake among HIV-positive women, it would not be erroneous to expect that a significant number of HIV-positive women are suffering from an additional burden of cervical cancer, which impacts their lifespan and quality of life. Recent studies have demonstrated that sexually transmitted diseases such as HIV/AIDS are more likely to be linked to CC than human papilloma virus [20]. Women infected with HIV are more likely to be diagnosed with cervical cancer than uninfected women [20–24]; hence, strategic intervention plans need to be implemented and scaled up for screening uptake and scheduled follow-up among HIV-positive women to detect precancerous lesions promptly.

We identified the following barriers that hinder this target group from undertaking the service: Knowledge about cervical cancer and screening, Risk perception of cervical cancer, Fear of test result and fear of screening as painful, Access to screening services, Cost of screening service, and Partner attitude and acceptance of the service.

In line with the findings reported by SSA countries on barriers to and facilitators of CC screening among the whole population[ 53, 54], we also identified that 49% of HIV-positive women had poor knowledge of cervical cancer and screening. In SSA countries, where there is significant illiteracy, there is a lack of information and knowledge about the high mortality rate from CC and the early control and screening mechanisms of the disease. Fear of test results and screening (18%) and risk of perception of the disease (23%) were the other barriers identified in this review. Poor knowledge more likely intensifies misconceptions about the disease and service, fear and sense of pain and low perceived risk of the disease. Individual challenges stated by HIV-positive women are that they do not want to go screening for the reason of stigma, they do not want to add another stress, and the burden of the disease scares them more than anything else.

Poor partner attitude and acceptance of the service were other reasons for not screening for CC among HIV-positive women. This finding is consistent with reports done in Uganda, [59], Iran, [60], and Britain, [61]. This might be explained by understanding that in most SSA countries, the head of the family and sole decision maker is often the husband, and depending on cultural practices, CC screening may be perceived as violating the woman’s privacy.

Our review demonstrated that a lack of access to screening services was a barrier to uptake. Difficulties in navigating health care facilities, lack of awareness about screening locations, and prolonged waiting times are all potential contributors to limited uptake. The other identified barrier was the high cost of the service. This finding is in line with findings reported from the United States [62], Nigeria [63], and Tanzania [64]. In areas where poverty prevails significantly, payment for nonemergency health services such as CC screening is unlikely. Furthermore, the cost of transportation is a huge barrier in choosing to use or not use a CC screening service. Overall, this review demonstrated that the perception of an additional burden of having a cervical cancer diagnosis appears to be a unique barrier among this population of women who are HIV positive. Although it is expected that this group of women comes into contact with the care system on a regular basis, poor counselling of physicians and poor coordination of services to cervical cancer screening contribute to the low coverage of cervical cancer screening among these groups of populations. High heterogeneity as a result of differences in health care systems, access to healthcare, screening context and study design is visible across the countries; however, factors that may account for the heterogeneity from the same country in similar healthcare settings might be due to the same screening context, including but not limited to a difference in the tools for the measurement of uptake of cervical cancer screening, context of screening (mass/clinic based) and difference in sample size. Interventions addressing the above barriers could help increase the uptake of cervical cancer screening service.

### Implication of this review

This review adds further evidence of low coverage of cervical cancer screening among women living with HIV/AIDS in SSA countries, despite significant association between HIV/AIDS and cervical cancer. This implies a further need of promotional policies, enhanced nationwide advocacy, strengthening of health education and creation awareness programs through media outlets, integrating of cervical cancer screening to primary health care levels and to routine treatments among these vulnerable and high risk group of women. It also shows that, though this group of women is expected to come into contact with the care system on regular basis, poor medical advice, and poor coordination of cervical cancer screening services to routine treatment contributes to a low level of cervical cancer screening among these groups.

### Limitations

This finding should be interpreted considering the limitations of the study. High heterogeneity as a result of differences in health care systems, implementation of services between countries, high discrepancy in uptake of cervical cancer screening, and underrepresentation of countries may affect the generalizability of the review to all Sub-Saharan countries. However, the concern of heterogeneity was addressed using a random effect model for pooling the estimates. As a limitation, all the included articles only measured lifetime receipt of cervical cancer screening; however, it is possible that HIV-positive women may have been screened before they tested positive.

Although we included unpublished papers, there might still be unpublished articles concerning this topic.

## Conclusion

The unacceptably low coverage of uptake of cervical cancer screening would indicate that the need to scale up the opportunities to these groups of women as well. This review revealed that in addition to structural and health care system barriers, sociocultural and personal barriers are powerful barriers in HIV-positive women. For these cohorts of population, a particular obstacle was discovered to be perception of an additional burden of having cervical cancer.

## Supplementary Information

**Additional file 1: Annex Table S1.** Newcastle - Ottawa quality assessment scale for cohort stduy designs.

**Additional file 2: Annex 2 Table S2.** Newcastle - Ottawa quality assessment scale for cross-sectional stduy designs.

## Data Availability

Data and materials are available at any time with the main author at email address meresaberu@gmail.com.

## Abbreviations

CC: Cervical cancer
HIV: Human immunodeficiency virus
SSA: Sub Saharan Africa
WHO: World Health Organization
