# Supplementary material for: Uptake and barriers to cervical cancer screening among human immunodeficiency virus-positive women in Sub Saharan Africa: a systematic review and meta-analysis
Source: BMC Womens Health. 2023 Jun 27;23:338. doi: 10.1186/s12905-023-02479-w (PMC10294344; doi:10.1186/s12905-023-02479-w)
Supplement: Supplementary file 1 — Additional file 1: Annex Table S1. Newcastle - Ottawa quality assessment scale for cohort stduy designs. [file 12905_2023_2479_MOESM1_ESM.docx]

**Annex Table S1: NEWCASTLE - OTTAWA QUALITY ASSESSMENT SCALE FOR COHORT STDUY DESIGNS**

| Author, yr, reference | Checklist(NOS) | | | | | | | |
| --- | --- | --- | --- | --- | --- | --- | --- | --- |
|  | Representative of the exposed cohort | Selection of the non exposed cohort | Ascertainment of exposure | Demonstration that outcome of interest was not present | Comparability of cohorts | Adequacy of follow up of cohorts | Assessment of outcome | Total score  NOS |
| Preetam C. et al.2017([33](file:///C:\Users\user\Downloads\Edited%20Figures%20and%20Tables%20AJE.docx#_ENREF_33)) | 2 | 1 | 1 | 1 | 1 | 1 | 1 | 7 |
| Boris K. et al.2017([45](file:///C:\Users\user\Downloads\Edited%20Figures%20and%20Tables%20AJE.docx#_ENREF_45)) | 2 | 1 | 2 | 1 | 1 | 1 | 1 | 9 |
