# Supplementary material for: Uptake and barriers to cervical cancer screening among human immunodeficiency virus-positive women in Sub Saharan Africa: a systematic review and meta-analysis
Source: BMC Womens Health. 2023 Jun 27;23:338. doi: 10.1186/s12905-023-02479-w (PMC10294344; doi:10.1186/s12905-023-02479-w)
Supplement: Supplementary file 2 — Additional file 2: Annex 2 Table S2. Newcastle - Ottawa quality assessment scale for cross-sectional stduy designs. [file 12905_2023_2479_MOESM2_ESM.docx]

**Annex 2** Table S2:  **NEWCASTLE - OTTAWA QUALITY ASSESSMENT SCALE FOR CROSS-SECTIONAL STDUY DESIGNS**

| Author, yr,ref | **Checklist(NOS)** | | | | | | |
| --- | --- | --- | --- | --- | --- | --- | --- |
|  | **Representative of the sample** | **Sample size** | **Response rate** | **Ascertainment of the exposure (risk factor)** | **The subjects in different outcome groups are comparable** | **Description of Statistical test** | **Total score NOS(9)** |
| Belete N. et al.2015 ([34](file:///C:\Users\user\Downloads\Edited%20Figures%20and%20Tables%20AJE.docx#_ENREF_34)) | 2 | 1 | 2 | 1 | 1 | 1 | 8 |
| Ashagrie A. et al.2017([35](file:///C:\Users\user\Downloads\Edited%20Figures%20and%20Tables%20AJE.docx#_ENREF_35)) | 2 | 1 | 2 | 1 | 1 | 1 | 8 |
| kalkidan S. et al.2019([36](file:///C:\Users\user\Downloads\Edited%20Figures%20and%20Tables%20AJE.docx#_ENREF_36)) | 2 | 1 | 2 | 1 | 1 | 1 | 8 |
| Apollinaire H. et al.2012([46](file:///C:\Users\user\Downloads\Edited%20Figures%20and%20Tables%20AJE.docx#_ENREF_46)) | 0 | 2 | 2 | 1 | 1 | 1 | 7 |
| Nega A. et al.2018([37](file:///C:\Users\user\Downloads\Edited%20Figures%20and%20Tables%20AJE.docx#_ENREF_37)) | 2 | 1 | 2 | 1 | 1 | 1 | 8 |
| Assefa A. et al.2019([38](file:///C:\Users\user\Downloads\Edited%20Figures%20and%20Tables%20AJE.docx#_ENREF_38)) | 2 | 1 | 2 | 1 | 1 | 1 | 8 |
| Bugembe I.2019([47](file:///C:\Users\user\Downloads\Edited%20Figures%20and%20Tables%20AJE.docx#_ENREF_47)) | 2 | 1 | 1 | 1 | 1 | 1 | 7 |
| Jasintha M.2019([49](file:///C:\Users\user\Downloads\Edited%20Figures%20and%20Tables%20AJE.docx#_ENREF_49)) | 2 | 1 | 2 | 1 | 1 | 1 | 8 |
| Oliver C.etal.2013([48](file:///C:\Users\user\Downloads\Edited%20Figures%20and%20Tables%20AJE.docx#_ENREF_48)) | 2 | 1 | 2 | 2 | 1 | 1 | 9 |
| Mokhele I.etal.2016([50](file:///C:\Users\user\Downloads\Edited%20Figures%20and%20Tables%20AJE.docx#_ENREF_50)) | 2 | 1 | 1 | 2 | 1 | 1 | 8 |
| Rhoda K.etal.2017([31](file:///C:\Users\user\Downloads\Edited%20Figures%20and%20Tables%20AJE.docx#_ENREF_31)) | 2 | 1 | 2 | 2 | 1 | 1 | 9 |
| Judith L.etal.2017([43](file:///C:\Users\user\Downloads\Edited%20Figures%20and%20Tables%20AJE.docx#_ENREF_43)) | 1 | 1 | 2 | 1 | 1 | 1 | 7 |
| Tirivanhu C.etal.2013([30](file:///C:\Users\user\Downloads\Edited%20Figures%20and%20Tables%20AJE.docx#_ENREF_30)) | 0 | 0 | 0 | 1 | 1 | 1 | 3 |
| Olivia M.2014([44](file:///C:\Users\user\Downloads\Edited%20Figures%20and%20Tables%20AJE.docx#_ENREF_44)) | 1 | 1 | 1 | 1 | 1 | 1 | 6 |
| Saba S. et al.2017([40](file:///C:\Users\user\Downloads\Edited%20Figures%20and%20Tables%20AJE.docx#_ENREF_40)) | 2 | 1 | 2 | 1 | 1 | 1 | 8 |
| Leilt G.2018([39](file:///C:\Users\user\Downloads\Edited%20Figures%20and%20Tables%20AJE.docx#_ENREF_39)) | 2 | 1 | 2 | 1 | 1 | 1 | 8 |
| Daniel A. et al. 2017([41](file:///C:\Users\user\Downloads\Edited%20Figures%20and%20Tables%20AJE.docx#_ENREF_41)) | 1 | 1 | 2 | 1 | 1 | 1 | 7 |
| Kathryn E.2019([42](file:///C:\Users\user\Downloads\Edited%20Figures%20and%20Tables%20AJE.docx#_ENREF_42)) | 2 | 1 | 2 | 2 | 1 | 1 | 9 |
| Bulto G.etal.2019([32](file:///C:\Users\user\Downloads\Edited%20Figures%20and%20Tables%20AJE.docx#_ENREF_32)) | 2 | 1 | 2 | 1 | 1 | 1 | 8 |
